# Stress increases blood beta‐hydroxybutyrate levels and prefrontal cortex NLRP3 activity jointly in a rodent model

**DOI:** 10.1002/npr2.12164

**Published:** 2021-02-20

**Authors:** Tsuyoshi Nishiguchi, Masaaki Iwata, Naofumi Kajitani, Akihiko Miura, Ryoichi Matsuo, Shumei Murakami, Yumeto Nakada, Shenghong Pu, Yuki Shimizu, Tatsuya Tsubakino, Takehiko Yamanashi, Gen Shinozaki, Jun Tsubota, Yukihiko Shirayama, Ken Watanabe, Koichi Kaneko

**Affiliations:** ^1^ Faculty of Medicine Department of Neuropsychiatry Tottori University Yonago Japan; ^2^ Division of Clinical Laboratory Tottori University Hospital Tottori Japan; ^3^ Department of Psychiatry University of Iowa Carver College of Medicine Iowa City IA USA; ^4^ Energy Technology Laboratories Osaka Gas Co., Ltd. Osaka Japan; ^5^ Department of Psychiatry Teikyo University Chiba Medical Center Ichihara Japan; ^6^ Watanabe Hospital Tottori Japan

**Keywords:** beta‐hydroxybutyrate (BHB), depression, inflammasomes, leucine‐rich repeat, nucleotide‐binding domain, prefrontal cortex, pyrin domain‐containing 3 (NLRP3), stress

## Abstract

**Aim:**

This study aimed to assess the response of endogenous beta‐hydroxybutyrate to psychological stress, and its association with nucleotide‐binding domain, leucine‐rich repeat, pyrin domain‐containing 3 (NLRP3) inflammasome, and stress‐induced behavior.

**Methods:**

Male C57BL/6J mice were subjected to 1‐hour restraint stress to examine changes in the endogenous beta‐hydroxybutyrate and active NLRP3 levels in the prefrontal cortex. Subsequently, we created a depression model applying 10‐day social defeat stress to the male C57BL/6J mice.

**Results:**

One‐hour restraint stress rapidly increased beta‐hydroxybutyrate levels in the blood. The active NLRP3 levels in the prefrontal cortex also increased significantly. A correlation was found between the increased beta‐hydroxybutyrate levels in the blood and the active NLRP3 levels in the prefrontal cortex. The mice exposed to social defeat stress exhibited depression‐ and anxiety‐like behavioral changes in the open field, social interaction, and forced swim tests. There was a correlation between these behavioral changes and endogenous beta‐hydroxybutyrate levels. Among the social defeat model mice, those with high beta‐hydroxybutyrate levels tended to have more depression‐ and anxiety‐like behavior.

**Conclusions:**

The increased blood beta‐hydroxybutyrate levels due to psychological stress correlate with the active NLRP3 levels in the prefrontal cortex, suggesting that the increased beta‐hydroxybutyrate levels due to stress may reflect a reaction to brain inflammation. In addition, mice with higher blood beta‐hydroxybutyrate levels tend to exhibit increased depression‐ and anxiety‐like behaviors; thus, an increase in blood beta‐hydroxybutyrate levels due to stress may indicate stress vulnerability.

## Abstract 2

Psychological stress increased blood beta‐hydroxybutyrate (BHB) levels. Mice with higher blood BHB levels tend to exhibit increased depression‐ and anxiety‐like behaviors. The increased blood BHB levels correlate with the active NLRP3 levels in the prefrontal cortex, suggesting that increased blood BHB levels may represent stress vulnerability.

## Graphical abstract

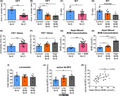

## 1 INTRODUCTION

Depression is characterized by a depressed mood, diminished interest or pleasure, fatigue or loss of energy, a feeling of worthlessness, or excessive or inappropriate guilt^1^. The pathophysiology underlying depression and the response to and outcome of its treatment are not yet fully understood. Various pathological hypotheses of depression represented by the monoamine hypothesis have been proposed, and the current pharmacotherapeutics for depression are based on these hypotheses. However, it is estimated that approximately 30%‐50% of depressed patients do not respond to antidepressants^2^. Therefore, various new hypotheses have been proposed to account for the contradictions to the conventional hypotheses, leading to a novel understanding of the pathophysiology of depression. In recent years, neuroinflammation has been drawing attention as one of the main factors involved in the development of mood disorders, such as depression^3^, ^4^, ^5^, ^6^, ^7^, ^8^. Psychological stress is a known risk factor for the development of depression^9^, ^10^, and human studies indicate that psychosocial stressors increase peripheral cytokine production, a potentially important factor in the development of depression or anxiety^11^. The adenosine triphosphate/purinergic type 2x7 receptor/nucleotide‐binding domain, leucine‐rich repeat, pyrin domain‐containing 3 (NLRP3) inflammasome cascade induces neuroinflammation due to psychological stress. This inflammation has been reported to play an integral role in depressive and anxious behaviors, suggesting its pathophysiologic role in depression^12^. In addition, NLRP3 inflammasome is activated in mononuclear blood cells of patients with major depressive disorder^13^, ^14^. Based on these discoveries, the inhibition of NLRP3 inflammasome activity and inflammatory substances downstream may provide an avenue to develop new treatments for depression. In recent years, reports have indicated that the administration of beta‐hydroxybutyrate (BHB), an endogenous ketone body, suppresses depressive behavior and inflammatory response resulting from psychological stress^10^. These studies suggest that BHB may be a potential therapeutic agent for psychiatric disorders, such as stress‐related mood disorders^15^. However, the relationship between NLRP3 inflammasome activity, BHB, and depression‐related behavior is yet to be established, and understanding its pathophysiology is of great interest.

In this study, we focused on the dynamics of stress‐induced endogenous BHB. We examined the effects of acute psychological stress on endogenous BHB levels and NLRP3 inflammasome activity in brain tissues. Numerous studies suggest that depression and anxiety in humans result in part from the hypoactivation and reduced volume of the frontal cortical and hippocampal regions that control subcortical structures such as the nucleus accumbens and amygdala, although hyperactivation of certain prefrontal cortex regions is also involved^16^. In postmortem studies of the prefrontal cortex in major depression, depressed subjects differed significantly from control subjects in several prefrontal cortical areas. They had decreased cortical thickness, neuronal size, and number of glial cells^17^. One study reported that microglial NLRP3 inflammasome activation mediates IL‐1b‐related inflammation in the prefrontal cortex of depressive rats^18^. Therefore, we especially focused on the prefrontal cortex in this study. We also used a social defeat (SD) model to induce depression‐ and anxiety‐like behaviors and analyzed their association with endogenous BHB levels and NLRP3 activity in the prefrontal cortex. BHB has been reported to be the blood metabolite most correlated with the severity of depression^19^. The purpose of this study was to clarify the role that BHB plays in depression.

## 2 METHODS

### 2.1 Animals

Male C57BL/6J mice (8‐9 weeks of age) and male CD‐1 mice (older than 10 weeks of age) were purchased from Charles River Laboratories (Yokohama, Japan). Four to five mice were housed per cage on a 12‐h light/dark cycle (lights on at 7:30 am). The temperature was maintained at 25°C, and food and water were freely available. One week before the experimental procedures, the mice were acclimatized to the laboratory.

### 2.2 Experimental procedures

#### 2.2.1 Experiment 1

**FIGURE 1:**
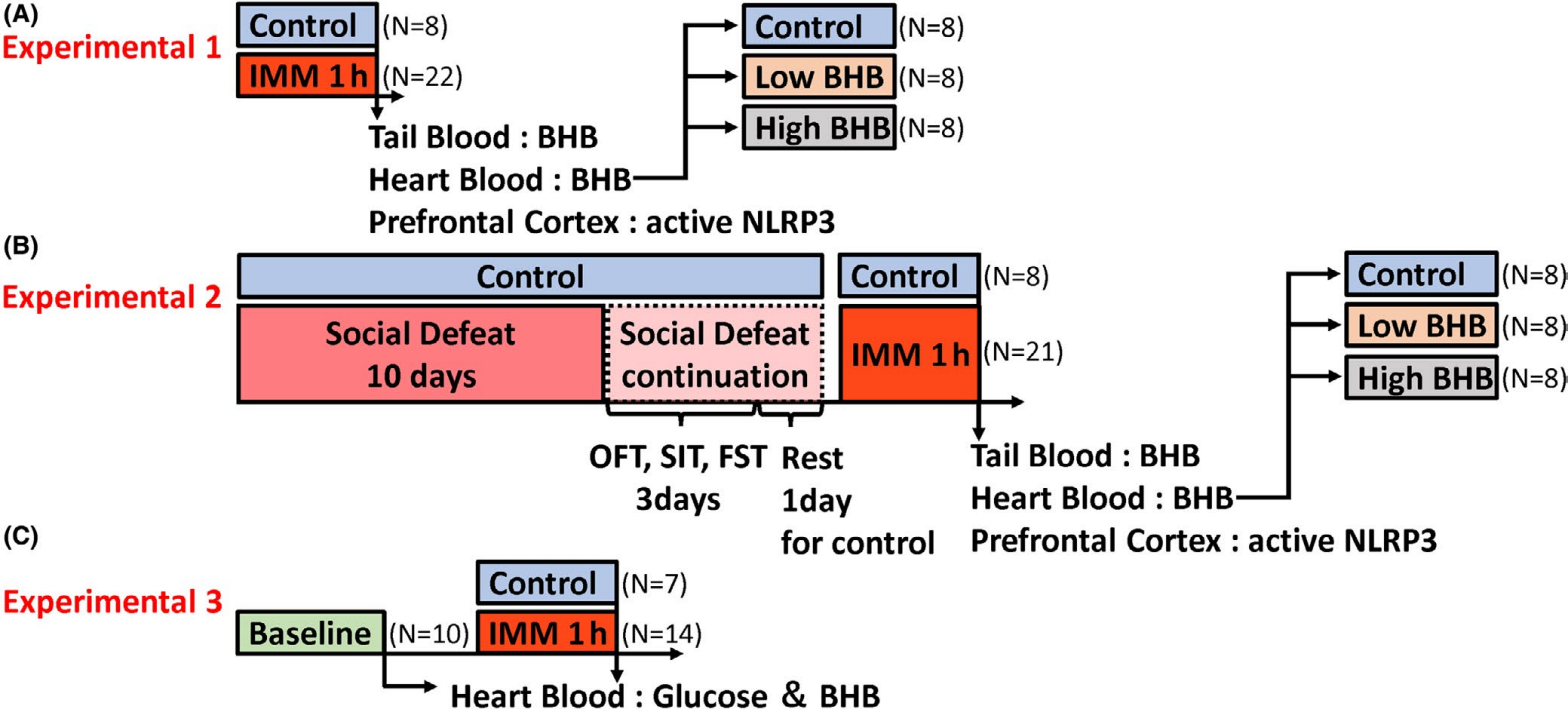
Experimental design. A, Male C57BL/6J mice were divided into a control group (n = 8) and an IMM group (n = 22), and the IMM group was subjected to 1 h of IMM stress. Subsequently, tail blood, heart blood, and prefrontal cortex of all mice were collected. B, Male C57BL/6J mice were divided into a control group (n = 8) and an SD group (n = 22), and the SD group was subjected to social defeat stress for 10 days. Subsequently, OFT, SIT, and FST were performed on all mice. Then, after a rest day, the SD group was subjected to IMM stress the next day, and the tail blood, heart blood, and prefrontal cortex of all the mice were collected. C, Male C57BL/6J mice were divided into a baseline group (n = 10), a control group (n = 7), and an IMM group (n = 14), and heart blood was first collected from the baseline group. Subsequently, the IMM group was subjected to restraint stress for 1 h, and then, heart blood was collected from the control and IMM groups. IMM, immobilization stress; BHB, beta‐hydroxybutyrate; OFT, open field test; SIT, social interaction test; FST, forced swim test

This experiment evaluated the BHB levels in blood and NLRP3 activity in the prefrontal cortex caused by acute stress. Thirty male C57BL/6J mice were prepared; eight as a control group and 22 as a 1‐hour immobilization (IMM) stress group were randomly allocated and were used in the experiment. The IMM was administered by putting a mouse into a 50‐mL tube. In the IMM stress group, the eight animals with the lowest blood BHB levels were defined as the low BHB group, and the eight with the highest levels, the high BHB group. Thus, the control, low BHB, and high BHB groups included eight animals each (Figure 1A). The remaining six mice were excluded from this experiment.

#### 2.2.2 Experiment 2

Thirty C57BL/6J mice were prepared, eight as a control group and the remaining 22 as an SD group were randomly allocated (one mouse was dead during the SD stress exposure). After 10 days of SD stress exposure, all mice were subjected to open field test (OFT), social interaction test (SIT), and forced swim tests (FST). The SD mice may have been stressed differently depending on the strength of the aggression from the preceding aggressor. Therefore, after a one‐day rest period, the SD group mice were subjected to the 1‐h IMM stress test to keep the stress conditions constant before blood collection. Tail and heart blood and brain tissue were collected immediately after the IMM stress test (Figure 1B).

#### 2.2.3 Experiment 3

Thirty‐one C57BL/6J mice were prepared: 10 in the baseline group, seven in the control group, and the remaining 14 in the IMM group were randomly allocated. The IMM group was subjected to 1‐h IMM stress. Heart blood was collected from the baseline group at 0‐h and from the control and IMM groups 1‐h later (Figure 1C).

#### 2.2.4 Social defeat mice model of stress‐induced depression

**FIGURE 2:**
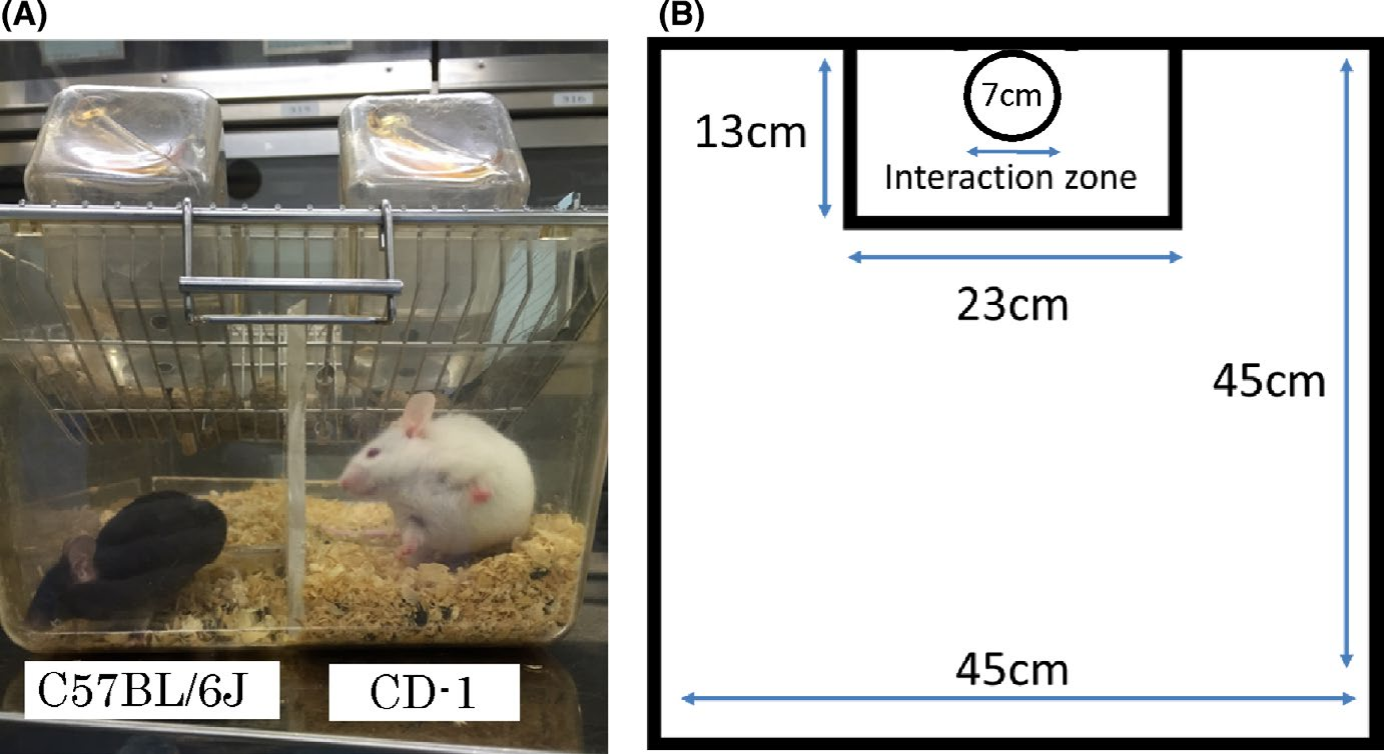
Social defeat stress and social interaction test. A, The mouse on the left is a C57BL/6J mouse, and the one on the right is a CD‐1 mouse. There is a transparent partition plate with a number of small holes between the two animals, through which they can visually and olfactorily recognize each other. B, Schematic diagram of the social interaction test. A mesh box with a diameter of 7 cm and a height of 10.6 cm in a box with a diameter of 45 cm and a height of 40 cm. The surrounding area of 13 × 23 cm is defined as an interaction zone. The box is empty in the first trial, and CD‐1 is in the second trial

The repeated SD stress procedure was designed as described previously with minor modifications.^20^ In brief, CD‐1 mice were screened by placing a screener C57BL/6J mouse directly into the home cage of the aggressor CD‐1 mouse. CD‐1 mice were selected for use as aggressors in subsequent repeated SD experiments if the latency to initiate aggression was less than 60 seconds. Intruder C57BL/6J mice were placed directly within the resident aggressor’s home cage compartment for 10 minutes. The intruder was transferred across the perforated divider to the opposite compartment, where it stayed for the remainder of the 24‐h period (Figure 2A). For each subsequent daily defeat, the intruder C57BL/6J was moved to a novel resident’s home cage compartment to prevent any habituation to the resident aggressor for 10 consecutive days. Control mice were housed in pairs and separated by a divider.

### 2.3 Behavioral tests

#### 2.3.1 Open field test (OFT)

C57BL/6J mice were placed in an open field apparatus (45×45×40 cm) and allowed to explore for 10‐min. The test was conducted in a dark environment. The tests were recorded, and the time spent in the center area (25×25 cm) was measured. The open field apparatus was cleaned after each trial.

#### 2.3.2 Social interaction test (SIT)

Before the SIT was conducted, all experimental mice were acclimated in the laboratory room for at least an hour. Subsequently, according to the protocol^20^, each C57BL/6J mouse was carefully placed in the center of the social field twice, either with an empty mesh cage (7 cm in diameter and 10.6 cm height, first 150‐s trial) or with a completely novel CD‐1 mouse in the enclosure (second 150‐s trial) (Figure 2B). The C57BL/6J mice were placed back in their own cages for a 30‐s rest period between the two trials. The C57BL/6J mice’s exploratory activity in the two trials was recorded and analyzed using a video tracking software system (SMART 3.0: Panlab, U.S.A.). For each mouse, the social interaction (SI) ratio was calculated as (interaction time, CD‐1 present)/(interaction time, CD‐1 absent) × 100%. Locomotion was also measured in the first 150‐s.

#### 2.3.3 Forced swim test (FST)

C57BL/6J mice were placed in a 3L beaker filled with water (24°C, 15 cm depth) for 10‐min. Immobility was defined as the point at which the mouse ceased struggling and remained in the water without movement. Tests were recorded and scored using a video tracking software system (SMART 3.0, Panlab, USA). The water was changed after each trial.

### 2.4 Measurement of BHB levels from tail vessels

C57BL/6J mice were anesthetized with isoflurane. The mice’s tail tips were cut by approximately 2 mm, and blood was collected from the cut surfaces. BHB levels were measured using the FreeStyle Precision Neo® (Abbott Laboratories, Abbott Park, IL, USA) according to the manufacturer’s standard protocol.

### 2.5 Blood and prefrontal cortex sample collection

C57BL/6J mice were anesthetized with isoflurane. The mice were thoracotomized, using a needle inserted directly into the right ventricle. Approximately 0.7 mL of blood was collected with a syringe containing EDTA, the blood was centrifuged at 3000 × g for 5 minutes, and the plasma was separated. The mice were decapitated, and the prefrontal cortex was collected.

### 2.6 Plasma BHB and glucose measurement

Precise plasma BHB levels were measured with LABOSPECT 006 (Hitachi, Ltd, Tokyo, Japan) using the Wako Autokit 3‐HB (FUJIFILM Wako Diagnostics, Lexington, MA, USA), which has high sensitivity and high specificity as it uses cyclic enzymatic reactions, according to the manufacturer’s standard protocol. Precise plasma glucose levels were measured with LABOSPECT 006 using the Aqua‐auto Kainos GLU (Kainos Laboratories Inc, Tokyo, Japan).

### 2.7 Co‐immunoprecipitation

The NLRP3 inflammasome was co‐immunoprecipitated using the Dynabeads® Co‐Immunoprecipitation Kit (Thermo Fisher Scientific, Waltham, MA, USA) according to the manufacturer’s instructions. Anti‐ASC/TMS1 antibody (Proteintech, Thermo Fisher Scientific, Waltham, MA, USA) was incubated with Dynabeads® M‐270. Anti‐ASC antibody was supposed to immunoprecipitate ASC, NLRP3, and Caspase‐1.

### 2.8 Western blotting

Western blot was conducted in our laboratory as previously described.^15^, ^21^ Briefly, frozen prefrontal cortex tissue samples were homogenized in an ice‐cold buffer. The protein samples were separated using 12% sodium dodecyl sulfate‐polyacrylamide gel electrophoresis and then transferred to PVDF membranes. The membranes were incubated with primary antibodies against NLRP3 (AG‐20B‐0014: 1:1000 dilution, Adipogen, San Diego, CA, USA), followed by incubation with an anti‐mouse secondary antibody (ab97023; 1:10 000 dilution, Abcam, Cambridge, MA, USA). The positive and negative controls for NLRP3 were obtained from OriGene (Rockville, MD, USA). Densitometric analysis of western blot bands was performed using ImageJ software, version 1.51 (National Institutes of Health, Bethesda, MD, USA).

### 2.9 Statistical analyses

All statistical analyses were conducted using statistical package for the Social Sciences Statistics 19.0 (Tokyo, Japan). Statistical differences were determined by analysis of variance followed by Tukey’s post hoc analysis (three groups) or Student’s *t* test (two groups). The data are presented as mean ± standard error of the mean. *P * < 0.05 were considered statistically significant.

## 3 Results

### 3.1 Mice exposed to acute stress show increased BHB levels collected from heart and tail vessels and increased NLRP3 activity in the prefrontal cortex

**FIGURE 3:**
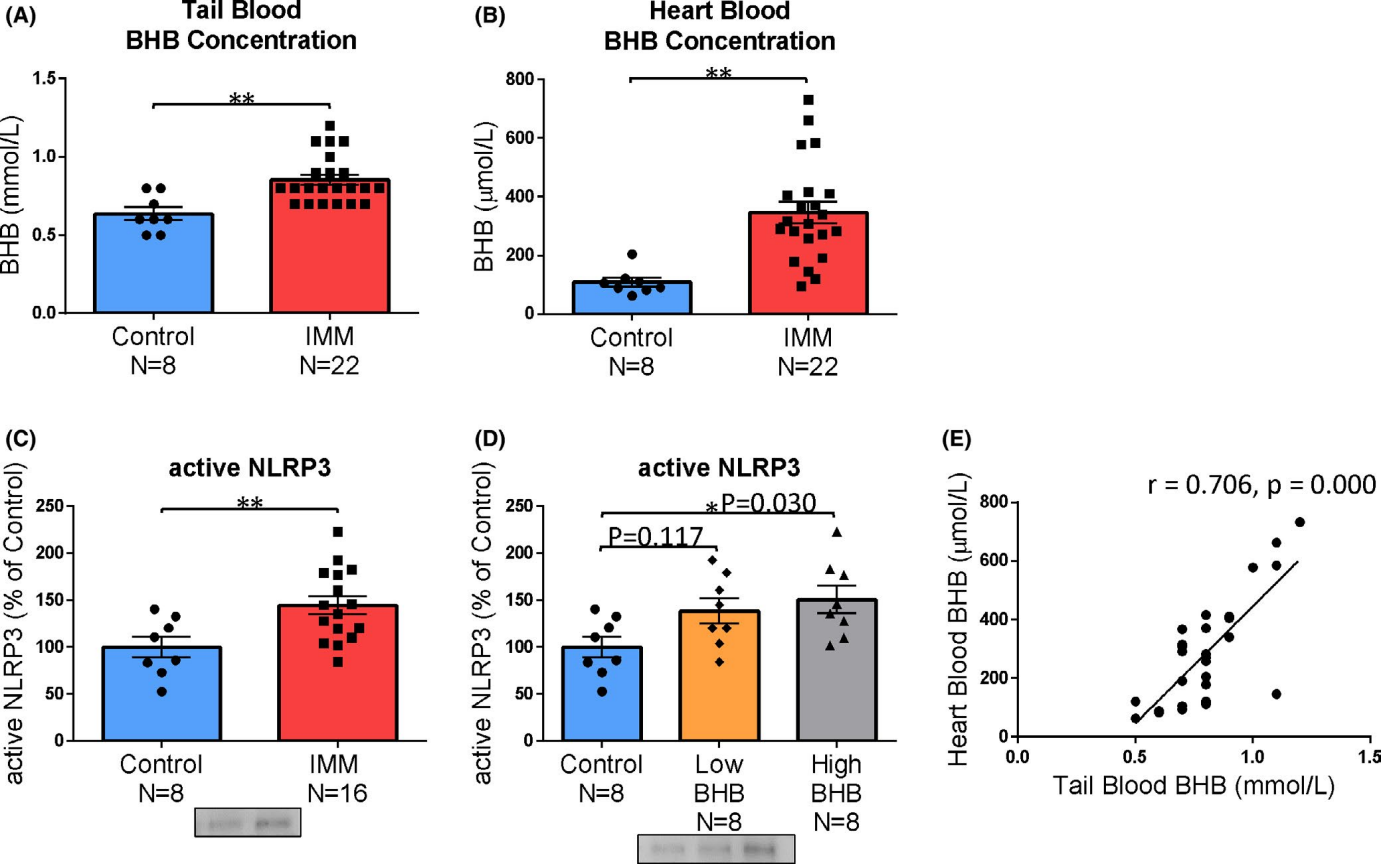
Acute stress increases blood BHB levels and NLRP3 activity in the prefrontal cortex. A, BHB levels in tail blood (simple measurement), B, BHB levels in heart blood (precise measurement), C, active NLRP3 levels in the prefrontal cortex, D, IMM group seen in C was divided into low BHB group and high BHB group. E, Accurately measured BHB levels in the heart blood and BHB levels measured by simple measurement in the tail blood were strongly correlated. Error bars represent the standard error of the mean (SEM). ^*^ *P *< 0.05 and ^**^ *P* < 0.01

After applying IMM stress for 1‐h, the BHB levels were measured in the tail blood (simple measurement) and heart blood (reference standard measurement). We found that the BHB levels were significantly increased in both tail blood (*t* = −3.612, *P = *0.001) and heart blood (*t* = −3.861, *P = *0001) after 1‐h of IMM stress (Figure 3A,B). In addition, 1‐h IMM stress significantly activated NLRP3 in the prefrontal cortex (*t* = 2.832, *P = *0.010) (Figure 3C). Regarding NLRP3 activity, when the low and high BHB groups were compared with the control group separately, we found a significant increase in the BHB levels only in the high BHB group (F [2,21] = 4.134, *P *= 0.031) (Figure 3D). BHB levels in heart blood and tail blood were strongly correlated (*r* = 0.706, *P* = 0.000) (Figure 3E).

### 3.2 Social defeat stress induces depression‐ and anxiety‐like behavior, especially in the high BHB group

**FIGURE 4:**
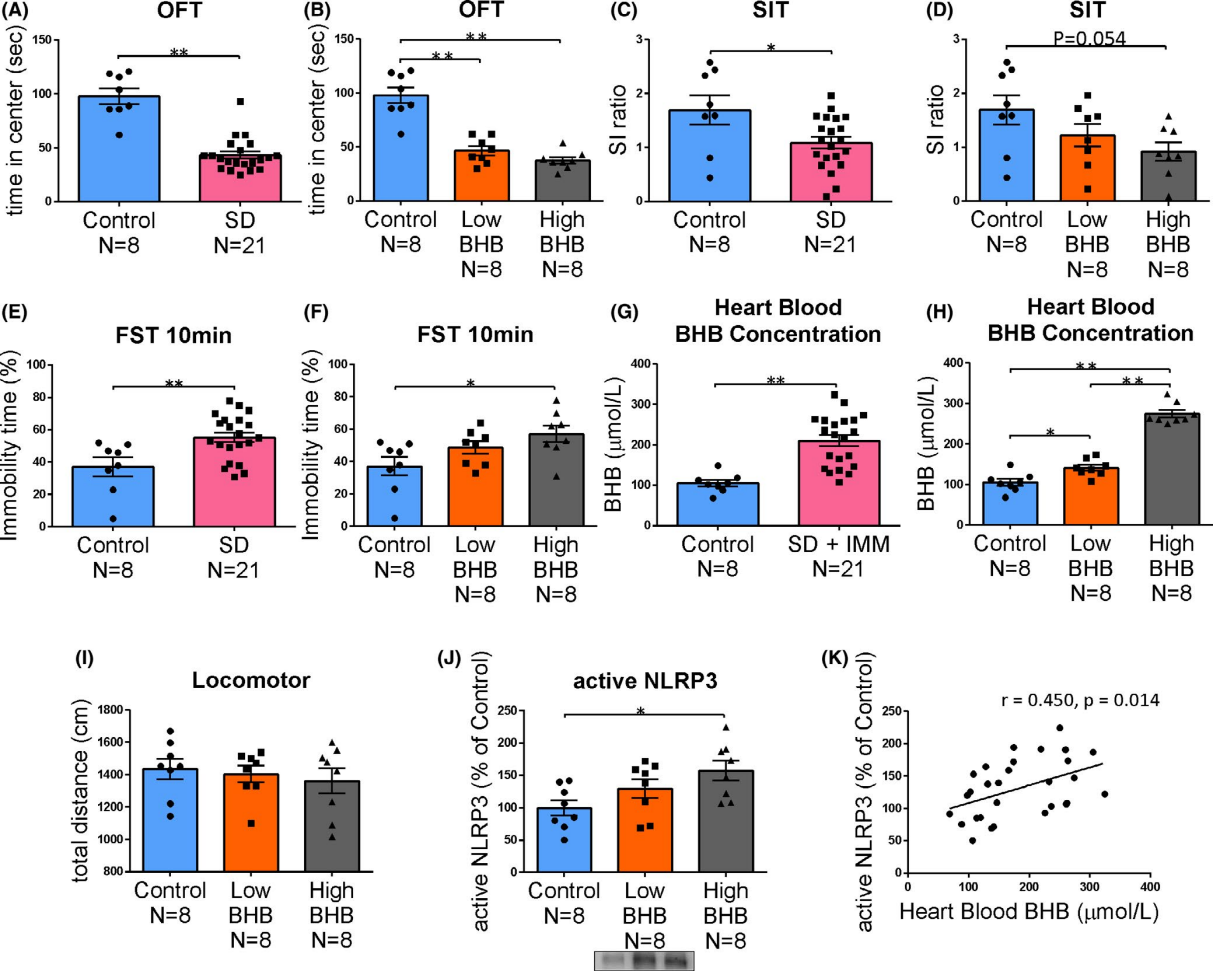
Behavioral test results after social defeat stress, blood BHB levels, and prefrontal cortex NLRP3 activity. After 10 days of social defeat stress, various behavioral tests were conducted. A and B, Time in the center zone in the open field test (OFT); C and D: Social interaction (SI) ratio in the social interaction test (SIT). E and F, Immobility time at 10 min in the forced swim test (FST). G and H, BHB levels in heart blood (precise measurement) after 1 h of IMM stress (SD group only). I, Locomotor activity measured in the SIT. J: Active NLRP3 levels after 1 h of IMM stress (SD group only). K, BHB levels in the heart blood and the active NLRP3 levels in the prefrontal cortex were moderately correlated. Error bars represent the standard error of the mean (SEM). ^*^ *P *< 0.05 and ^**^ *P* < 0.01

After 10 days of SD stress, various behavioral tests (OFT, SIT, and FST) were conducted. In the OFT, the time spent in the central zone was significantly reduced in the SD group (*t* = 7.755, *P* = 0.000) (Figure 4A), likely due to anxiety. IMM was performed to measure BHB levels in the heart blood (*t* = −4.478, *P* = 0.000) (Figure 4G). When low and high BHB groups were compared to the control group (Figure 4H), there were significant differences between the control group and the low BHB group (*P* = 0.000), and high BHB group (*P* = 0.000), which was not observed between the low and high BHB groups (*P* = 0.461) (F (2, 21) = 39.336, *P* = 0.000) (Figure 4B).

In the SIT group, the SI ratio was significantly lower in the SD group than in the control group (*t* = 2.520, *P* = 0.018) (Figure 4C), likely due to anxiety. When the SD group was divided into a low BHB group and a high BHB group, the SI ratio was lower in the high BHB group than in the control group (*P* = 0.054), but not in the low BHB group (*P* = 0.609) (F [2, 21] = 3.126, *P* = 0.065) (Figure 4D). Although there was no significant difference between the low and high BHB groups, the high BHB group had higher anxiety levels.

In the FST, the immobility time was significantly longer in the SD group than in the control group (*t* = −3.001, *P* = 0.006) (Figure 4E). From this, it seems that SD caused behavioral despair. When the SD group was further divided into a low BHB group and a high BHB group, the high BHB group exhibited significantly increased inactivity compared to the control group (*P* = 0.025). However, the low BHB group showed no significant difference from the control group (*P* = 0.244) (F [2, 21] = 4.112, *P* = 0.031) (Figure 4F). We inferred that the high BHB group exhibited behavioral despair. Since locomotor activity was not significantly different between the three groups (control vs low BHB; *P* = 0.937, control vs high BHB; *P* = 0.711, low BHB vs high BHB; *P* = 0.895) (Figure 4I), the change in FST does not appear to depend on the amount of exercise. The active NLRP3 levels in the prefrontal cortex of the control, low BHB, and high BHB groups showed the same trend as did the BHB levels in the heart blood (F [2, 21] = 4.251, *P* = 0.028) (Figure 4J). BHB levels in heart blood and the active NLRP3 levels in the prefrontal cortex were moderately correlated (*r* = 0.450, *P* = 0.014) (Figure 4K). Inflammation may influence the background of behavioral changes. However, no significant association could be perceived between active NLRP3 levels in the prefrontal cortex and behavior (data not shown).

### 3.3 Acute stress increases blood BHB levels without lowering blood glucose levels

**FIGURE 5:**
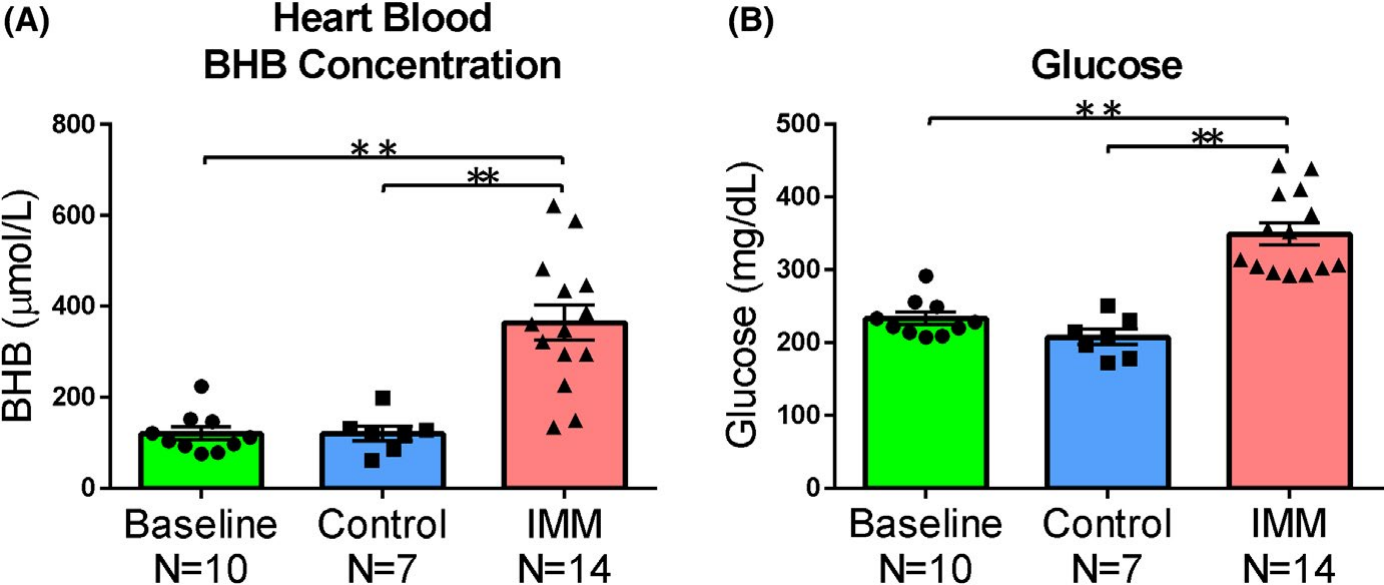
Acute stress increases the blood glucose level. A, BHB levels (precise measurement) in the heart blood. In the baseline group, blood was collected before IMM stress was applied; in the control group, blood was collected without 1 h of stress; and in the IMM group, blood was collected after 1 h of restraint stress. B, Blood glucose in the heart blood (precise measurement). In the baseline group, blood was collected before IMM stress was applied; in the control group, blood was collected without 1 h of stress; and in the IMM group, blood was collected after 1 h of restraint stress. Error bars represent the standard error of the mean (SEM). **P < 0.01

We conducted an additional experiment. Generally, BHB is known to increase during starvation.^22^ In this experiment, we investigated whether the increase in blood BHB levels induced by acute stress was due to decreased blood glucose. Blood BHB levels were similar in the control group compared to the baseline group but were significantly higher in the stress group (baseline vs control; *P* = 1.000, baseline vs IMM; *P* = 0.000, control vs IMM; *P* = 0.000) (F [2.28] = 34.149, *P* = 0.000) (Figure 5A). Blood glucose levels were also unchanged in the control group but were elevated, rather than decreased, in the stress group (baseline vs control; *P* = 0.465, baseline vs IMM; *P* = 0.000, control vs IMM; *P* = 0.000) (F [2, 28] = 21.086, *P* = 0.000) (Figure 5B).

## 4 Discussion

IMM has been shown to increase the expression level of active NLRP3^12^. It has also been reported that BHB administration suppresses NLRP3 inflammasome activity and has antidepressant‐like effects^15^, ^21^, ^23^, ^24^. Thus, our prediction before the current study was that the active NLRP3 expression level would be reduced in the group with high blood BHB levels. Also, it was thought that the blood BHB levels might be an index of stress resilience. However, in experiment 1, we confirmed that IMM rapidly increased the endogenous blood BHB levels, and when the IMM group was divided into the low and high BHB groups, and the relationship with the active NLRP3 expression level was investigated, the high BHB group exhibited a significantly higher active NLRP3 expression level than the control group. However, this difference was not significant in the low BHB group. Therefore, BHB levels may be increased in response to neuroinflammation, and this increase may result in a correlation with NLRP3, which is an index of neuroinflammation. Thus, the increase in endogenous BHB levels may reflect the degree of neuroinflammation.

If the increase in endogenous BHB reflects neuroinflammation, how is it related to depressive behavior? In experiment 2, after investigating depressive behavior under chronic stress due to SD, the increase in BHB levels and the expression level of active NLRP3 due to IMM were examined in the same manner as in experiment 1; how stress‐induced BHB elevation is related to NLRP3 levels and behavior was also examined. In experiment 2, as in experiment 1, the IMM group was divided into a low and a high BHB group, and the relationship with the active NLRP3 expression level was investigated. The high BHB group, but not the low BHB group, was significantly more depressive in FST than the control group (Figure 4F). The expression level of NLRP3 was also high in this group, but there was no significant difference in the low BHB group relative to the control group (Figure 4J).

With depressive behavior, the expression level of active NLRP3 was significantly higher in the high BHB group than in the control group. However, there was no significant difference in the low BHB group. In addition, a correlation was found between the increase in BHB levels and the increase in active NLRP3 expression level. These results suggest that endogenous BHB levels may be elevated in response to neuroinflammation and may be reflective of depressive behavior. Because of their reactivity, it is thought that the dynamics of endogenous BHB alone do not suppress depression‐related behaviors. One study indicated that activation of NLRP3 plays a pivotal role in the development of depression‐ and anxiety‐like behavior and in the cellular and molecular alterations associated with depression^25^. Moreover, it has recently been reported that BHB might exert its anti‐inflammatory effects in a rodent depression and post‐traumatic stress disorder model via NLRP3 inflammasome modulation^15^, ^21^, ^26^. Therefore, we believe that the prophylactic and therapeutic effects of exogenous BHB administration may result in new therapeutic agents for depressive conditions.

By contrast, in the low BHB group, despite being stressed, a predominant difference was observed only in the behavior in OFT. However, there was no difference from control in other active NLRP3 expression and depression‐ and anxiety‐like behaviors, which may reflect stress resistance. When grouped according to BHB levels, we were able to distinguish differences in stress tolerance between the low and high BHB groups (data not shown). On the contrary, when separated from the difference in stress tolerance by behavioral tests, no correlation with the difference in BHB levels could be observed. Therefore, stress toleration/vulnerability factors are not known in this study, but it was suggested that the endogenous BHB levels are an index of vulnerability.

A study whose results differ from ours showed that the levels of BHB in the brain reduced during the inflammatory state^27^. One possible explanation for this difference is that the blood BHB dynamics may differ from the brain BHB dynamics. Because very little glycogen is stored in the brain, the high energy requirements of the central nervous system must be met by external sources such as glucose and BHB^28^, ^29^. It is known that BHB functions as an alternative energy substrate in the brain, but it is synthesized in the liver of mammals^30^, ^31^. Thus, BHB may be increased in blood and used as fuel in brain during stress and an inflammatory state. However, the BHB levels in brain were not measured in this study.

In recent years, it has been reported that the blood BHB levels of depressed patients reflect the severity of depression^19^. In mice, the same pattern was observed. These results suggest that BHB levels are associated with the depression pathophysiology and are mediated by stress‐induced neuroinflammation. Therefore, it is desirable to verify the dynamics of inflammatory substances in brain tissue, but this experiment has not been able to assess that question. In the future, we plan to study the pathophysiology associated with the dynamics of inflammatory substances in depressive states related to psychological stress.

It is safe to measure BHB levels using a relatively minimally invasive blood test. In this experiment, a simple measuring device showed excellent correlation with a test using a blood obtained from heart, and it is less invasive. It has been suggested that endogenous BHB can be a safe diagnostic marker for depression or various other mental disorders in the future.

In this study, we found a correlation between the increase in BHB levels in the blood caused by acute stress and active NLRP3 in the prefrontal cortex. However, no clear correlation was found between the active NLRP3 levels in the prefrontal cortex and SD‐induced depression‐ and anxiety‐like behaviors. In this study, depression‐ and anxiety‐like behaviors due to SD stress were correlated with BHB levels in the blood. Because of the above results, the increase in BHB may not necessarily respond only to the activation of NLRP3 (inflammation). Further studies are needed on the triggers for increased BHB levels due to stress.

In conclusion, acute stress increased blood BHB levels. The changes were associated with active NLRP3 levels in the prefrontal cortex and depression‐ and anxiety‐like behaviors, indicating that increased blood BHB levels may represent stress vulnerability.

## 5 ANIMAL STUDIES

The experimental procedures were conducted in accordance with the Institutional Animal Care Guidelines and were approved by the Tottori University Animal Care and Use Committee (Approval number h31‐Y012). Efforts were made to minimize animal suffering.

## CONFLICT OF INTEREST

The authors declare no conflict of interest.

## AUTHOR CONTRIBUTIONS

TN and MI conceived the study and participated in its design and coordination. TN, MI, NK, AM, RM, SM, YN, YS, and TT performed the experiments. SP and MI analyzed the data. TN and MI wrote the manuscript. TY, GS, JT, YS, KW, and KK critically reviewed the manuscript.

## Supporting information

Table

## Data Availability

The data that supports the findings of this study are available in Data S1 of this article.
